# Electrical Stimulation to Enhance Wound Healing

**DOI:** 10.3390/jfb12020040

**Published:** 2021-06-19

**Authors:** Saranya B. Rajendran, Kirsty Challen, Karen L. Wright, John G. Hardy

**Affiliations:** 1 Lancaster Medical School, Faculty of Health and Medicine, Lancaster University, Lancaster, Lancashire LA1 4AT, UK; s.b.rajendran@lancaster.ac.uk; 2 Emergency Department, Lancashire Teaching Hospitals NHS Trust, Royal Preston Hospital, Sharoe Green Lane, Preston, Lancashire PR2 9HT, UK; kirsty.challen@lthtr.nhs.uk; 3 Division of Biomedical and Life Sciences, Faculty of Health and Medicine, Lancaster University, Lancaster, Lancashire LA1 4YG, UK; 4 Department of Chemistry, Faculty of Science and Technology, Lancaster University, Lancaster, Lancashire LA1 4YB, UK; 5 Materials Science Institute, Lancaster University, Lancaster, Lancashire LA1 4YB, UK

**Keywords:** wound healing, chronic wounds, electrical stimulation, cell biology

## Abstract

Electrical stimulation (ES) can serve as a therapeutic modality accelerating the healing of wounds, particularly chronic wounds which have impaired healing due to complications from underlying pathology. This review explores how ES affects the cellular mechanisms of wound healing, and its effectiveness in treating acute and chronic wounds. Literature searches with no publication date restrictions were conducted using the Cochrane Library, Medline, Web of Science, Google Scholar and PubMed databases, and 30 full-text articles met the inclusion criteria. In vitro and in vivo experiments investigating the effect of ES on the general mechanisms of healing demonstrated increased epithelialization, fibroblast migration, and vascularity around wounds. Six in vitro studies demonstrated bactericidal effects upon exposure to alternating and pulsed current. Twelve randomized controlled trials (RCTs) investigated the effect of pulsed current on chronic wound healing. All reviewed RCTs demonstrated a larger reduction in wound size and increased healing rate when compared to control groups. In conclusion, ES therapy can contribute to improved chronic wound healing and potentially reduce the financial burden associated with wound management. However, the variations in the wound characteristics, patient demographics, and ES parameters used across studies present opportunities for systematic RCT studies in the future.

## 1. Introduction

Our skin is our body’s largest external organ and it plays a crucial role in acting as the first line of defense against mechanical and pathogenic threats, and ideally, any injury to this barrier will be repaired rapidly [1].

**Figure 1:**
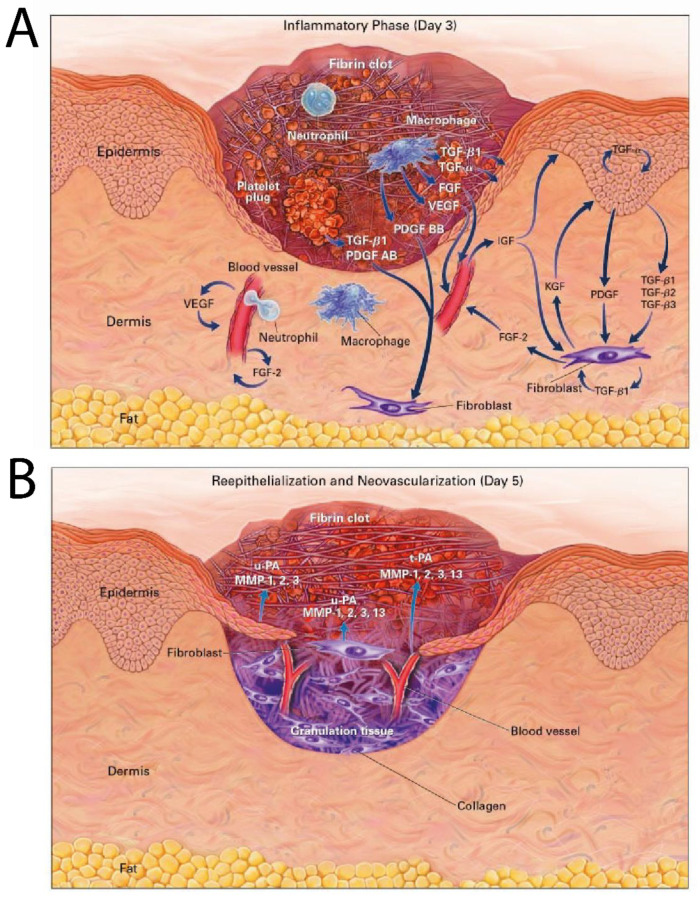
(**A**) A cutaneous wound 3 days after injury. Growth factors thought to be necessary for cell movement into the wound are shown. TGF-β1, TGF-β2, and TGF-β3 denote transforming growth factor β1, β2, and β3, respectively; TGF-α transforming growth factor α; FGF fibroblast growth factor; VEGF vascular endothelial growth factor; PDGF, PDGF AB, and PDGF BB platelet-derived growth factor, platelet-derived growth factor AB, and platelet-derived growth factor BB, respectively; IGF insulin-like growth factor; and KGF keratinocyte growth factor. (**B**) A cutaneous wound 5 days after injury. Blood vessels are seen sprouting into the fibrin clot as epidermal cells resurface the wound. Proteinases thought to be necessary for cell movement are shown. The abbreviation u-PA denotes urokinase-type plasminogen activator; MMP-1, 2, 3, and 13 matrix metalloproteinases 1, 2, 3, and 13 (collagenase 1, gelatinase A, stromelysin 1, and collagenase 3, respectively); and t-PA tissue plasminogen activator. Images reproduced with permission from [4].

A wound is any injury causing a break in the structure of living tissue, which may go beyond the skin’s epithelial layer to affect the underlying subcutaneous structures depending on the extent of damage [1]. Wound healing is a complex yet well-orchestrated physiological process involving a variety of cells and chemical mediators. The series of events involved in wound healing can be broadly classified into three main phases: (a) The inflammatory phase, (b) the proliferative phase, and (c) the remodeling phase [1]. The events occurring in these phases involve hemostasis to control bleeding, migration of inflammatory cells to the wound site (chemotaxis), granulation tissue formation, collagen repair, vascularization, and re-epithelialization [2]. These important events work through a signaling system coordinated by a myriad of mediators such as growth factors and cytokines [2]. Examples of these include transforming growth factor (TGF), insulin-like growth factor (IGF), fibroblast growth factor (FGF), vascular endothelial growth factor (VEGF), keratinocyte growth factor (KGF), and platelet derived growth factor (PDGF), that collectively help induce differentiation of immune cells to clear debris and fight infection, stimulate growth, promote formation of new blood vessels, and release inflammatory mediators [3]. Figure 1 depicts the stages of cutaneous wound healing (specifically Figure 1A depicts a wound during the inflammatory phase of healing and Figure 1B depicts a wound during the proliferative and remodeling phase of healing) and the respective growth factors released to stimulate immune cells and cutaneous structures.

**Figure 2:**
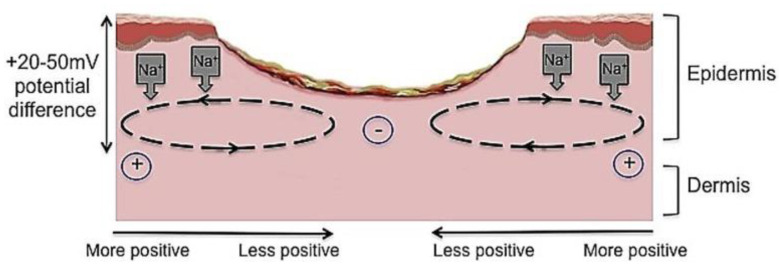
The current of injury is thought to be significant in initiating repair. Undamaged human skin has an endogenous electrical potential and a transcutaneous current potential of 20–50 mV. This is generated by the movement of sodium ions through Na+/K+ ATPase pumps in the epidermis. The current of injury is generated through epithelial disruption. Following an injury to the skin, a flow of current through the wound pathway generates a lateral electrical field and this is termed the “current of injury” or “skin battery” effect. Image reproduced with permission from [11].

The wound healing process is also influenced by our skin’s endogenous electric potential [5], also dubbed the endogenous “skin battery” [6]. In undamaged skin, a natural electrical potential of 10–60 mV between the epidermal and sub-epidermal layer exists [6]. This is largely attributed to the transport of ions through ion channels and the frequent depolarization and repolarization of cells [7]. This trans-epithelial voltage (TEP) largely increases around a wound. The disruption to the epithelium by an injury creates a short-circuit to the TEP, driving positive electrical flow towards the wound, as depicted in Figure 2. [8,9]. Injuries produce an electric current [10], and clinical studies have shown that the voltage difference between the wound site and the undamaged skin ranges between 100 and 150 mV/mm [7,8,9].

These endogenous electric fields play a critical role in wound healing [7,8], with resulting endogenous currents acting as a cue for cellular migration which concomitantly help heal wounds [8]. In addition, it is noteworthy that without this current, it is estimated that the average healing rate decreases by 25% [12]. This phenomenon motivates the exploration of the use of electrical stimulation (ES) to accelerate wound healing for various applications [13].

Most cutaneous lesions take a week or two to heal. However, this is prolonged in chronic wounds, which do not progress systematically through the healing stages [14]. This can be due to factors that hamper the wound healing process such as age, obesity, smoking, nutritional deficiencies or underlying diseases that predispose patients to develop chronic wounds (e.g., diabetes mellitus and/or peripheral venous disease) [14]. In conditions such as diabetes, wounds remain in a chronic inflammatory phase due to impaired cellular migration, growth factor release, and poor microcirculation [15]. In addition to this, chronic wounds host various microbes that colonize and multiply within the unhealed tissue, further contributing to impaired healing [15].

**Figure 3:**
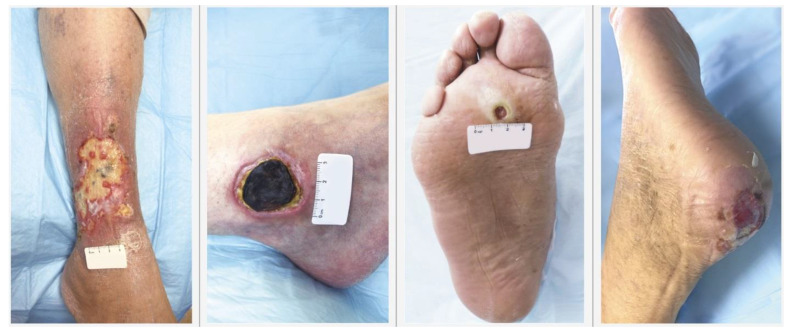
Types of chronic wounds (from left to right): Venous leg ulcer, arterial leg ulcer, neuropathic diabetic foot ulcer, and pressure ulcer. Image adapted with permission from [20].

Chronic wounds broadly include diabetic ulcers, pressure sores, and ulcers caused by arterial and venous insufficiency (vascular ulcers) [16]. Figure 3 depicts these chronic wounds and their pathophysiology is briefly described in Table 1 [17]. The staging of pressure and diabetic foot ulcers is outlined in Appendix A. Some researchers postulate that the endogenous current observed upon injury is markedly reduced in chronic wounds, contributing to its impaired healing [18]. Although these wounds have different etiologies, they possess common characteristics including: Excessive inflammation, tendency to get recurrent infection, improper vascularization, and slower migration of epithelial cells to mediate repair [17,18,19].

Chronic wounds predominantly affect older patients and with the steady rise in the elderly population worldwide, its incidence continues to grow [21]. As of 2013, the annual cost of managing chronic wounds in the United Kingdom’s National Health Service (NHS) was roughly GBP 3.2 billion [22]. According to a health report from 2008, more than 400,000 people in the UK have chronic wounds, with venous leg ulcers being the most common [23]. These non-healing wounds impair the patient’s quality of life, and pose a burden on the NHS limited resources [17,18,19].

The therapeutic use of electrical stimulation in medical practice has been established particularly in pain and wound management. Some international clinical guidelines published by bodies such as the Australian Wound Management Association and the Consortium for Spinal Cord Medicine 2014 suggest the use of ES to help promote chronic wound healing [24,25]. The parameters to the ES can be adjusted to varying frequencies, wave amplitude, duration of exposure, and pulse type. The application of ES is typically painless and commonly administered by placing electrodes around the wound, which then deliver short bursts of electrical potential which results in electrical currents [26]. ES can be administered to chronic and acute wounds in various ways: Using electrodes placed around the wound (most common method, e.g., POSiFECT^®^ medical device on a pressure ulcer located on the sacral region [12]), electro-biofeedback application of ES using a device with electrodes placed on different sites around the wound, bioelectric dressings applied to a wound site, wireless application of ES to the wound site [11]. Although typically painless, users report experiencing paresthesia or a tingling sensation around the area of application [26]. Commonly used ES in medical practice and their features are described in Table 2 [11,27].

This review focuses on our current understanding of how ES influences the cellular mechanisms involved in normal cutaneous wound healing, its antibacterial effects, and the clinical effectiveness of ES in accelerating chronic wound healing.

## 2. Literature Search Method

Literature searches with no publication date restriction were conducted in four databases: The Cochrane Library, MedLine, PubMed, Web of Science and Google Scholar. The search strategy included the following terms: “Electric* stimulation”, “wound healing”, “cutaneous OR skin wound”, and “chronic wound”. “Randomized controlled trial” was included for chronic wound studies. The terms were merged with the Boolean operator “AND”. Titles and abstracts of published studies were subsequently reviewed.

The inclusion criteria were: Full text records, in vitro experiments on human tissue, randomized controlled trials, primary research papers, and studies on cutaneous and chronic wounds only. We excluded duplicate studies, non-English papers, studies unrelated to wound healing or chronic wound healing and unclear ES specifications. To assess the impact of ES on the normal process of wound healing, in vitro studies and randomized controlled trials (RCTs) on human skin are included. The second aim of this review focused on the effects of ES on chronic wound healing, for which only in vivo RCTs on human participants are included.

## 3. Results

The literature search yielded 30 studies meeting the inclusion criteria. Findings are categorized into studies that investigate the effects of ES on bacteria colonizing wounds, the effects of ES on the stages of healing, and clinical trials that investigate its effect on chronic wounds.

### 3.1. Effects of ES on Bacteria Colonising Wounds

Six in vitro studies investigated the bacteriostatic effects of exogenous ES [28,29,30,31,32,33]. Petrofsky et al. [28] exposed *Staphylococcus aureus*, *Pseudomonas aeruginosa*, and *Escherichia coli* to alternating current (AC) or direct current (DC). These three bacteria commonly colonize open wounds. The authors reported that 100 µA of DC had no inhibitory effect on any of the bacterial strain. In addition, 20 mA of AC significantly reduced the colony-forming unit (CFU) of only *P. aeruginosa* by 38% (*p* < 0.05). In contrast, Kincaid and Lavoie [29] noted that the growth of all three bacteria were inhibited at the anode and cathode when exposed to HVPC for 2 h at 250 V or more. Focusing on chronic wounds, Gomes et al. [30] exposed these three strains obtained from chronic venous ulcers to fixed diphasic (FD-B) current and high voltage monophasic current (HVMC) for 30 and 60 min, respectively. FD-B totally inhibited the colony forming unit (CFU) of all three strains in 30 min (*p* < 0.05). However, HVMC led to an average 50% CFU reduction for all but the *E. coli* strain.

In an in vitro study, Daeschlein et al. [31] demonstrated the antibacterial effect of electric current on six bacteria that commonly colonize chronic wounds. They experimented on three Gram-negative species (*E. coli*, *P. aeruginosa*, *Klebsiella pneumonia*) and three Gram-positive species (*S. aureus*, *Staphylococcus epidermidis*, *Escherichia faecium*). Exposure to 42 mA of monophasic low voltage pulsed current (LVPC) for 30 min resulted in a significant reduction in CFU in all tested pathogens (*p* < 0.01), whereas no antibacterial effects were noted in the control group. They also noted that applying positive polarity to the current had greater bacteriostatic effects than negative polarity. Barranco et al. [32] exposed *S. aureus* to DC using different electrodes (silver, platinum, gold, and steel) and varying currents of 0.4, 4, 40, and 400 µA for 48 h, they reported that the positive silver anode resulted in the highest reduction in bacterial growth at a lower current range (0.4 and 4 µA). Falcone and Spadaro [33] also reported that the electrically active silver anode showed pronounced bacteriostatic effects even at a low direct current (4 µA). Asadi and Torkaman summarize the antibacterial effects of ES as a combination of direct effects (disruption of bacterial membranes and blocking the proliferation of bacterial cells) and indirect effects (pH changes, i.e. increased pH at the cathode and decreased pH at the anode; temperature changes; electrolysis products, e.g., toxic species, chlorine, H_2_O_2_, radicals; and galvanotaxis, increased migration of white blood cells such as macrophages and leukocytes to the infected wound site) [34].

There is a lack of studies investigating the antibacterial effects of electrical stimulation in vivo. Further in vivo research may be required to ascertain that exogenous ES can help limit the burden of infection accompanying dermal wounds clinically. However, current evidence favors the likelihood that ES can be used as an adjunct for infection control in wound therapies.

### 3.2. Effects of ES on Cellular Migration and Tissue Perfusion

The inflammatory phase of wound healing involves control of bleeding, removal of pathogens, and increased tissue perfusion to recruit cells [1]. It has been observed that cells involved in wound healing carry a charge and migrate towards an electric field with the opposite polarity [35,36]. For instance, macrophages migrate towards the anode, fibroblasts migrate towards the cathode, and neutrophils migrate to both the anode and cathode [36], this phenomenon is known as Galvanotaxis [7,36]. This was demonstrated via an experiment whereby participants’ skin was scarred and exposed to exogenous ES. An assessment of the cell composition in the skin exudate 6 h post-treatment showed that the neutrophil count was 24% higher in the exposed group than the control group due to the increased electrotaxis of human cells in an electric field [37].

Four randomized controlled studies and one case series looked into the effects of exogenous ES on tissue perfusion [38,39,40,41,42]. Cramp et al. [38] investigated the application of transcutaneous electrical nerve stimulation (TENS) to understand its effect on skin blood flow. After 15 min of exposure, skin perfusion was roughly four times higher using low-frequency TENS compared to high-frequency TENS. Clover et al. [39] noted an increase in capillary density by 25% and transcutaneous oxygen tension by 1.24-fold when treated with localized sub-contractile electrical stimulation for 6 weeks (whereas no significant increase was observed in the control group). The immediate improvement in cutaneous perfusion was also demonstrated by Peters et al. [40] who noted a significant increase in transcutaneous partial pressure of oxygen (TcPO_2_) by 27% in patients with peripheral arterial disease using direct current, compared to no change in TcPO_2_ in the control group. The effects of improvement were noted within 5 min of ES exposure.

In an interesting case series by Goldman et al. [41], the investigators instructed six patients with critically ischemic limbs (defined as TcPO_2_ < 10 mmHg) to use the high voltage pulsed current (HVPC) (80–330 V) along with their standard wound dressing to treat their wounds. They defined a TcPO_2_ below 20 mmHg as unfavorable for wound healing. Pre-exposure means that TcPO_2_ around wound edges were 2 ± 2 mmHg. Following 40 days of daily ES, the mean TcPO_2_ rose to 33 mmHg and wounds closed thereafter. In a subsequent RCT, Goldman et al. [42] continued to explore this by randomizing eight subjects with ischemic limb wounds to receive active or sham HVPC for 14 weeks. After 4 weeks, wounds treated with active HVPC decreased in size (*p* < 0.05) while wounds in the control group increased in size. By week 8, HVPC treated wounds had an average TcPO_2_ measuring 27 ± 12 mmHg, no longer at an ischemic range, whereas the control group had an average of 2 ± 2 mmHg. Laser Doppler measures indicated a 3-fold increase in dermal capillary perfusion in the treatment group (*p* < 0.01). Their trial demonstrated how electrotherapy (mainly HVPC) improves dermal microcirculation and wound healing. Table 3 summarizes the key findings of papers investigating the effect of ES on this phase of wound healing.

### 3.3. Effects of ES on the Proliferative and Remodelling Phase of Healing

Events that occur in these phases include vascularization by angiogenesis, fibroblast proliferation, granulation tissue formation, and re-epithelialization. Two in vivo RCTs [43,44] obtained punch biopsies from human volunteers and treated them with degenerate wave current (DW) to understand which mechanisms of healing are induced. In a double-blind prospective trial by Sebastian et al. [43], volunteers had small biopsies taken from each arm but DW was given to only one arm, allowing the participants to serve as their own control. The histological analysis showed that on average, DW-exposed arms expressed vascular endothelial growth factor (VEGF) 42% more than unexposed skin. On day 14, expression of endothelial cells was 6% higher, and RNA transcripts of type IV collagen was 8% higher than the control. In a RCT reported by Ud-Din et al. [44] with a similar study design, treating biopsy wounds with DW showed a 38.9% reduction in wound volume and a 78.6% increase in blood flow to the wound site compared to the control. The histological analysis indicated an increase in granulation tissue area and an average 35% rise in VEGF production. Both studies showed an upregulation of anti-inflammatory genes. Zhao et al. [45] also demonstrated how ES induces angiogenesis. Their in vitro experiment showed that 1.5–2.9 mV DC-treated endothelial cells produced VEGF 2.6 times more than the control group.

With regards to the remodeling stage, Rouabhia et al. [46] found that fibroblasts exposed to 200 mV/mm of DC quickly migrated from both ends of a sample wound, and the wound had fully closed in 24 h. However, over 30–40% of the wound area in a similar sample remained unhealed in the control group. There was also a significant increase in the production of fibroblast growth factor (FGF-1 and FGF-2) (*p* < 0.01). Snyder et al. [47] similarly identified an increase in random migration of fibroblasts by DC stimulation in their study. Cheng and Goldman [48] demonstrated that electrical exposure induces human dermal fibroblasts to enter into the growth phase of the cellular cycle. This possibly explains the stimulation of fibroblast proliferation upon ES exposure.

Sebastian et al. [49] reported that ES-treated wounded skin had a 38% increase in keratinocyte proliferation and an 18% increase in epithelium thickness compared to the control (*p* = 0.002). In contrast, Bullock et al. [50] found no significant change to keratinocyte proliferation when subjecting in vitro skin wound models to the pulsed current. In summary, the growth factors noted to be enhanced upon ES across the reviewed studies are FGF-1, FGF-2, and VEGF. These collectively aid in the proliferation of endothelial cells, fibroblasts, and expedites new vessel formation [51]. Table 4 summarizes key findings of in vivo and in vitro studies that investigate the effect of ES on this phase of healing [43,44,45,46,47,48,49].

### 3.4. Effects of ES on Chronic Wounds

This review evaluates 12 randomized controlled trials that assesses the effects of electrical stimulation (ES) on chronic wound healing [52,53,54,55,56,57,58,59,60,61,62,63]. The trials dated from 1988 to 2018, and included 532 participants. The studies used different types of ES such as HVPC (n = 8), PC (n = 3), and AC (n = 1) with varied parameters, and worked on different chronic wounds (pressure ulcers n = 6, diabetic ulcers n = 4, and vascular ulcers n = 2). The main challenges in interpreting the data were variation in the duration of treatment and measure of outcome. The primary outcome of measure used by most studies (n = 10) in quantifying the healing rate is the change in wound surface area measured as a percentage of area reduction (PAR) before and after the treatment. One study by Baker et al. [60] used weekly healing rate as an outcome, and a study by Griffin et al. [54] used percentage of wounds healed. A study by Polak et al. [63] assessed the peri-wound blood flow alongside PAR as their primary outcomes. Of the 12 studies, only four report both percentage of complete wound healing and changes in wound surface area [53,56,61,62].

In all studies, participants with chronic wounds were randomized to receive electrical stimulation therapy or sham treatment. Given that most trials took place in a clinical setting, participants also received standard wound care alongside ES or sham therapy. Two studies [59,62] used an electric stimulation device for patients to self-administer at home after receiving training. Of all trials, the study by Miller et al. [59] was the only study funded by a device manufacturer. Acknowledging the risk of bias, this was included in the review with interest in the results after noting that investigators initiated the research and abided by RCT protocols. Overall, all studies report a degree of accelerated chronic wound healing upon ES exposure.

Some trials included secondary measures of outcome such as changes in tissue perfusion, adverse effects of ES exposure, and the effect of participants’ compliance on wound healing. Lawson et al. [57] investigated whether blood flow to chronic wounds is increased with exogenous ES exposure. They report that tissue perfusion around the wounds increased by 87% in patients with diabetes and only 6% in patients without diabetes. Three studies reported adverse effects following ES exposure. In the study by Lawson et al. [57], two patients were hospitalized following unspecified complications and one dropped out after experiencing vertigo. Lundeberg et al. [61] found that 6% of ES group and 3% of sham experienced allergic symptoms, and 9% of ES group and 6% of sham group experienced pain. Similarly, Peters et al. [62] reported that five subjects (10% of ES and 15% of placebo group) dropped out following infection complications. It is uncertain whether the reported adverse effects can be fully attributed to ES exposure. Confounding factors include the severity of the subjects’ underlying disease. Of all studies, Peters et al. [62] were the only investigators who also reported whether compliance to the treatment affected the healing of wounds. Their results showed that 71% of ulcers healed in patients who were compliant, whereas only 50% of ulcers healed in the non-complaint group. Patients were classed as compliant if the ES exposure was >20 h/week. Table 5 summarizes the key findings of all 12 RCTs.

## 4. Discussion

### 4.1. General Considerations

This review aims to understand how electrical stimulation (ES) influences the normal processes involved in wound healing, followed by its efficacy in healing chronic wounds. In vitro experiments on human cells and tissues were included in this review in order to understand the mechanism of action of ES on a cellular level. In terms of the general activities of wound healing, reviewed in vivo and in vitro studies demonstrate that ES increases tissue perfusion, promotes cellular migration, increases tissue vascularization, and causes a significant improvement in fibroblast proliferation.

The reviewed experiments demonstrate increments in VEGF and FGF production upon ES exposure. ES has been shown to have a promising ability to accelerate wound healing by activating the angiogenesis signaling pathways and stimulating fibroblast proliferation. One in vitro study found that ES activates the mitogen-activated protein kinase (MAPK) signaling pathway, which triggered angiogenesis in the process of wound healing. Bai et al. [64] in a study on human endothelial cells found that ES induces VEGF receptor signaling, consequently upregulating VEGF secretion and endothelial cell migration. However, the underlying mechanism driving these molecular changes and increase in growth factors remain largely unclear.

Although an in vitro experiment [50] showed no improvement in re-epithelialization by keratinocytes upon PC stimulation, two others found improved re-epithelialization upon DW and PC stimulation [45,49]. The difference in wound type and ES used could account for that disparity. The selected studies reported p-values below 0.05, delineating the strong likelihood of ES contributing to enhanced healing. Findings from animal studies [36,65,66] further support this. Gürgen et al. [65] demonstrated that exposing rat wounds to transcutaneous electrical nerve stimulation (TENS) resulted in a significant reduction in pro-inflammatory cytokines. They also reported an overall reduction in wound healing time. Chu et al. [66] exposed guinea pig skin models to weak anodal DC (20–40 µA) to investigate its effect on wound healing. The authors report that DC treated wounds had less granulation tissue and fibrosis compared to control wounds. Other animal studies also report increased fibroblast proliferation and collagen deposition in ES treated wounds, which favors the remodeling phase of wound healing [36].

Six in vitro studies investigated the antibacterial effect of ES. Bacterial infection hinders wounds from healing quickly due to the excessive release of toxins and inflammatory signals. Chronic wounds have a larger risk of infection due to the prolonged healing time, making patients susceptible to septicemia [19]. Bacteria that commonly colonize acute and chronic wounds include *S. aureus*, *P. aeruginosa*, and *E. coli*. All six studies noted a reduction in the growth of at least one or all these bacterial strains when exposed to electricity. Three studies interestingly noted how a positive polarity using the silver anode showed marked bacteriostatic effects when exposed to a weak DC compared to the other electrode materials (likely due to the formation of reactive oxygen species). The pulsed current lead to a marked colony forming unit (CFU) reduction across three studies [29,30,31]. One mechanism behind this is that supplying electricity alters the pH of the bacteria’s environment. This consequently damages the external membrane of bacteria, allowing an uncontrolled influx of solutes that ultimately kill them [30]. Whilst the antibacterial effects of ES (that can be achieved using disinfection techniques), is beneficial for wound healing, supporting the potential use of ES in chronic wound treatment to minimize the clinical burden of infection.

Furthermore, the results of this review also indicate how ES, mainly HVPC, may enhance chronic wound healing upon regular administration. Secondary outcomes such as wound perfusion and complete wound healing were also positive upon ES exposure. Most trials utilized the pulsed current in their treatment (*n* = 11), with a majority of them using HVPC (n = 8). A meta-analysis of different ES on chronic wounds by Khouri et al. [67] reported that when comparing DC, LVPC, HVPC, and DW, HVPC showed the best improvement in chronic wound size reduction. Most in vivo trials that were reviewed used the pulsed current and reported positive outcomes. Unlike continuous DC, which has been noted to cause skin irritation due to pH changes, the monophasic pulsed current does not cause skin changes [27]. Furthermore, PC has polarity and is better able to mimic the physiological current in comparison to sinusoidal AC, which lacks polarity. These factors, along with the ability of PC to penetrate deeper into the skin partially explains why the pulsed current has been commonly used in the trials reviewed. However, it is difficult to ascertain a recommended method of ES application for chronic wounds as studies utilized varying parameters of ES and duration of exposure.

### 4.2. Limitations on Chronic Wound Studies

There are external factors that influence chronic wound healing such as age, pre-existing medical conditions, wound care method, and nutrient deficiency. Only one RCT was exclusively mentioned to have controlled these variables during their study [58]. In addition, it was the only study that followed up on their participants to assess full recovery. Only three reviewed studies assessed the adverse effects of treatment [57,61,62]. Long-term follow up, users’ perspectives and implications of ES on the participant’s quality life are lacking in most studies, as their primary measure of outcome was wound size reduction. Furthermore, the authors did not assess any extra burden caused by ES exposure to the patients, such as device-related complications and its acceptability. This limitation needs addressing in future trials if ES becomes a widespread treatment.

Although all considered RCTs demonstrated that ES markedly reduces chronic wound size, it is difficult to recommend an optimal plan using ES as a standard treatment. Although the pulsed current indicated significant success in accelerating chronic wound healing across all the trials that used it, differences exist in the polarity, duration, and method of ES exposure used across the studies. In addition, the demographics and wound characteristics of chosen participants varied across trials. This brings questions such as what is the ideal anatomical location and the optimal method of delivering ES? The variety in treatment protocols across trials makes it difficult to ascertain the most effective form of ES on a type of chronic wound. Another limitation is that in studies where participants had multiple co-morbidities, the extent to which those diseases contributed to impaired wound healing were not outlined.

### 4.3. Potential Implications for Clinical Practice

Chronic wound care consumes almost 3% of healthcare expenditure in developed countries [21]. In the United States, treating chronic venous leg ulcers cost USD 4000 per month per patient and advance wound dressings incurred additional costs up to USD 29,252 per treatment episode in 2012 [68]. Globally, the average cost of chronic wound care was USD 2.8 billion in 2014, and it is predicted to rise to USD 3.5 billion in 2021 [69]. A clinical study found that using ES therapy in adjunct with standard care reduces the costs associated with managing chronic wounds in the UK by 16% when compared to using standard procedures alone [70]. Another model indicated a potential reduction in NHS wound care costs by 15% if patients with chronic venous ulcers replaced their regular treatment with ES therapy [71]. The results of this review indicate that electrical stimulation increases the rate of chronic wound healing. More studies need to be conducted to investigate the effect of ES with time to complete chronic wound closure.

The current management of chronic wounds in the NHS include using advanced wound dressings that control moisture levels, antimicrobial dressings, negative pressure therapy, and offloading to reduce the pressure on wounds [72,73]. The National Institute for Health and Care Excellence (NICE) provide evidence-based recommendations for health care in England. The management of each type of chronic wound differs but the NICE guidelines recommend using proper wound dressing techniques to optimize quicker healing with minimal risk of infections [72]. Alternative therapies include skin grafting, growth factor therapy, hyperbaric oxygen therapy, and stem cell therapy. However, their reliable efficiency in chronic wound management have yet to be proven significant [73].

At present, the NICE guidelines do not recommend using ES therapy on chronic wounds unless it is used in a clinical trial [74]. It is suggested that ES should not be given to patients who have a pacemaker inserted, who have skin conditions, who are pregnant or patients with skin cancers and epilepsy [24]. The underlying health conditions of chronic wound patients should be taken into critical consideration. These limitations, followed by a lack of long-term therapeutic evidence on chronic wounds could suggest why ES is not used in regular practice in the UK. Contrastingly, physicians in Australia, New Zealand, and the United States are recommended to use ES as an adjunct therapy to treat chronic pressure ulcers [24,75]. The Pan Pacific Guideline for the Prevention and Management of Pressure Injury recommends the use of pulsed electrotherapy as an adjunct treatment to accelerate the healing of pressure ulcers [24]. Its use in other forms of chronic wounds such as diabetic ulcers is not specified.

## 5. Conclusions

Our bodies generate natural endogenous electrical potentials around a wound which are known to accelerate the healing process by guiding cells to migrate to the site of injury [8]. Exogenous electrical stimulation is used to mimic this physiological occurrence and it has been proven beneficial in accelerating wound healing. This review evidences that electrical stimulation limits inflammation, increases wound blood perfusion, controls bacterial growth, increases fibroblast migration, induces angiogenesis, and encourages keratinocyte activity. This applies to both acute and chronic wounds. Additionally, electrical stimulation (notably pulsed current) significantly reduces the size of chronic wounds when compared to control groups with no ES.

Current evidence supports the possibility of using ES as an adjunct therapy to chronic wound management. However, the most effective ES cannot be concluded from this review due to variations in the studies’ experimental protocol. Additionally, trials on chronic wounds did not compare different ES to suggest an ideal type. Future trials can compare different ES and monitor any long-term complications to recommend a specific type that contributes to the most effective healing.

We also foresee the potential of electroactive biomaterials to play a role in advanced wound healing technologies [76,77]. Recent advancements with nanogenerator technology producing self-sustainable ES as a wearable wound-healing device suggest the potential for exciting new opportunities in wound care management [78,79].

## Figures and Tables

**Table 1:** Types of chronic wounds and their underlying pathology.

Chronic Wound	Pathophysiology
Pressure ulcer	Necrosis of skin and underlying soft tissue due to prolonged unrelieved pressure, compression or friction.
Venous ulcer	Most commonly caused by venous hypertension due to faulty valves that lead to a sudden backflow of blood and increased pressure on vessel walls.
Arterial ulcer	Ischemic ulcers caused by obstructions that narrow arterial lumen such as embolisms or atheroma.
Diabetic ulcer	Commonly affects the lower extremities of the body. Loss of sensation (diabetic peripheral neuropathy), and existing vascular conditions contribute to foot ulcers.

**Table 2:** Characteristics of commonly used waveforms for electrical stimulation therapy.

Type of Exogenous ES	Characteristics
Direct current (DC)	Continuous flow of electric charge in a monophasic waveform (in one direction). Currents of 20–200 µA can be supplied at a low voltage.
Alternating current (AC)	Has a biphasic waveform, with two symmetrical electrical pulses alternating one after the other. Voltages typically 50–150 V dependent on tissue hydration.
Pulsed current (PC)	Intermittent flow of charged particles with gaps in current flow. This can have a monophasic or biphasic waveform. Currents of 1.2–1.5 mA can be supplied to the tissue at high voltage.
Degenerate wave (DW)	A type of waveform used in certain biofeedback devices. A constant current of 0.3 mA, which delivers an electric field of 10 mV/mm between the electrodes can be used.

**Table 3:** Key findings of studies investigating the effect of ES on skin perfusion.

Study Design	Type of ES	Exposure Duration	Experimented on	Key Outcome(s)	Reference
RCT	TENS	15 min	30 healthy subjects High frequency (n = 10) Low frequency (n = 10) Control (n = 10)	Increase in skin perfusion	[38]
RCT	Subcontractile ES	60 min/day Total 6 weeks	36 patients with ischemic limbs ES group (n = 24) Control (n = 12)	Increase in capillary density and skin perfusion	[39]
RCT	HVPC	Four 60-min periods Total 1 day	11 diabetic patients with and without PVD	Transient increase in TcPO_2_ in participants with PVD within 5 min	[40]
Case series	HVPC	1 h/day, 7 days/week for 1–9 months	6 patients with ischemic leg ulcers	Increased TcPO_2_ and total healing of ulcers post exposure	[41]
RCT	HVPC	1 h/day, 7 days/week, 14 weeks	8 Ischemic limb wounds ES group (n = 4) Sham treatment (n = 4)	Decreased wound size and increased peri-wound circulation	[42]

**Table 4:** In vitro and in vivo studies on the effects of exogenous ES on the proliferative and remodeling stages of wound healing.

Study Design	Type of ES	Exposure Duration	Experimented on	Key Outcome(s)	Reference
RCT	DW	14 days	20 healthy subjects, served as own control	Increase in VEGF, collagen, epidermal cells, and cell apoptotic markers	[43]
RCT	DW	14–20 days	40 healthy subjects, served as own control	Reduced wound volume, increased perfusion and vascularity	[44]
In vitro	Pulsed DC	At 4, 8, and 24 h	Human umbilical vein endothelial cell cultures	Increase in endothelial cell migration and VEGF production	[45]
In vitro	DC	At 2, 4, and 6 h	Human fibroblast cells in a wound model	Increase in FGF and differentiation of fibroblasts	[46]
In vitro	DC	10 min	Fibronectin coated and non-fibronectin coated dermal fibroblast cells	Increased random migration of fibroblast cells, no increase in dermal fibroblast gene expression	[47]
In vitro	AC	12 h	Dermal cell matrix	Dermal fibroblasts entered into the growth phase of cell cycle with continuous ES exposure	[48]
In vitro	DC and DW	16 days	Punch biopsies on sample human skin tissues	Increase in epidermis thickness and keratinocyte proliferation	[49]

**Table 5:** RCTs investigating the impact of electrical stimulation on chronic wound healing.

Study Design	Type of ES/Electrode Placement	Exposure Duration	Type of Chronic Wound	No. of Participants	% Wound Area Reduction/% of Wounds Healed	Reference
RCT	HVPC ^†^/Treatment electrode placed over wound	50 min/day, 5 days/week for 6 weeks	Pressure ulcers	63 patients Cathodal (n = 23) Anodal-cathodal (n = 20) Sham treatment(n = 20)	PAR ^1^ 82.34% and 70.77% in ES group, respectively, 40.53% in control/Wound healing not specified	[52]
RCT	HVPC/Treatment electrode placed over wound	50 min/day, 5 days/week for 6 weeks	Pressure ulcers	77 patients ES (n = 24) Sham (n = 28) US ^1^ (n = 25)	PAR 76.19% in ES group 48.97% in control group/52% of ulcers healed in ES group, 23% healed in control	[53]
RCT	HVPC/Treatment electrode placed over wound	60 min/day for 20 days	Pressure ulcers	17 patients ES (n = 8) Sham (n = 9)	Wound PAR not specified, higher in ES than control group/38% of ES wound healed vs. 22% in sham group (*p* > 0.05)	[54]
RCT	HVPC/Treatment electrode placed over wound	45–120 min daily for 5 weeks (45 min for sham treatment)	Pressure ulcers	60 patients ES (n = 45) Sham (n = 15)	After 60 and 120 min exposure PAR 91% in ES group vs. 25% in sham group/Wound healing not recorded	[55]
RCT	HVPC/Treatment electrode placed over wound	45 min/day, five days/week for average 7.4 weeks	Pressure ulcers	16 patients ES (n = 9) Sham (n = 7)	100% wound area reduction in treatment group, 28.9% increase in wound area in control group/Complete healing in ES group	[56]
RCT	Biphasic current/Treatment electrode places across wound on intact skin	30 min/day, 3 days/week, for 4 weeks	Mixed ulcers (diabetic and vascular)	17 patients Diabetic (n = 8) Non-diabetic (n = 9)	PAR 70% in diabetic group, 38.4% in non- diabetic group/Wound healing not specified	[57]
RCT	HVPC/Treatment electrode placed over wound	45 min/day, 3 days/week for 4 weeks	Mixed ulcers (diabetic and venous)	27 patients ES (n = 14) Sham (n = 13)	Wound PAR 44.3% in ES group and 16.6% in control group/Wound healing not specified	[58]
RCT	PC (using bodyflow device)/Treatment electrodes placed above and below the wound site	20 min/day, 4 days/week for 8 weeks	Venous ulcers	23 patients ES (n = 14) Sham (n = 9)	PAR 32.67% in ES, Sham ES 23.15%/Wound healing not recorded	[59]
RCT	Biphasic PC/Treatment electrodes placed over intact skin proximal to the wound site	30 min of exposure	Diabetic footulcers	80 patients Asymmetrical PC (n = 21), Symmetrical PC (n = 20), Low stimulation current (n = 19) Sham (n = 20)	Healing rate per week: 27% in asymmetrical PC, 16% in symmetrical PC, ~9.7% in control group/Wound healing not recorded	[60]
RCT	AC ^‡^/Not specified	20 min twice daily for 12 weeks	Diabetic footulcers	51 patients ES (n = 24) Placebo (n = 27)	PAR 61% in ES group, 41% in placebo group/42% of ES exposed ulcers healed vs. 15% in placebo group	[61]
RCT	HVPC/Treatment electrodes embedded in stockings placed around the wound	8 h nightly for 12 weeks	Diabetic footulcers	40 patients ES (n = 20) Sham (n = 20)	PAR 86% in ES group, 71% in sham group/65% of ES wounds healed vs. 35% of wounds in sham group	[62]
RCT	HVPC/Treatment electrodes placed on opposite edges of the wound site	50 min/day, 5 day/week for 8 weeks	Pressure ulcers	61 patients Anodal HVPC (n = 20) Cathodal HVPC (n = 21) Sham (n = 20)	PAR 64.1% in anodal HVPC group, 74.06% in cathodal HVPC group, 41.42% in sham group/Complete wound healed not recorded	[63]

^†^ HVPC: High voltage pulsed current; ^‡^ AC: Alternating current; ^1^ PAR: Percentage area reduction; US: Ultrasound.

## Data Availability

Not applicable.

## Appendix A

The stages in severity of diabetic and pressure ulcers are outlined in Table A1 [80] and Table A2 [81].

**Table A1:** Stages of pressure ulcers to assess severity in clinical practice.

Stage	Description of Wound
Stage I	Skin is intact with no open tears. Reddened area with possible blanching and pain. Area has a different texture compared to surrounding skin.
Stage II	Skin tears, forming an open wound with blisters. Painful with exudate release.
Stage III	Painful deep wound. Dermis is lost, fully exposing the fatty layer below. No muscle or bone visible.
Stage IV	Deeper, painful wound which exposes the underlying muscle, tendon, or bone.

**Table A2:** The University of Texas Staging System for diabetic foot ulcers.

Stage	Grade 0	Grade I	Grade II	Grade III
A	Ulcerative lesion completely epithelialized	Superficial ulcer, not penetrating to tendon or muscle	Ulcer penetrating to tendon or capsule	Ulcer penetrating to bone or joint
B	Infection	Infection	Infection	Infection
C	Ischemia	Ischemia	Ischemia	Ischemia
D	Infection and Ischemia	Infection and Ischemia	Infection and Ischemia	Infection and Ischemia
